# Finite Element Simulation and Sensitivity Analysis of the Cohesive Parameters for Delamination Modeling in Power Electronics Packages

**DOI:** 10.3390/ma16134808

**Published:** 2023-07-04

**Authors:** Giuseppe Mirone, Raffaele Barbagallo, Giuseppe Bua, Guido La Rosa

**Affiliations:** 1 Department of Civil Engineering and Architecture, University of Catania, Viale A. Doria 6, 95125 Catania, Italy; gmirone@dii.unict.it (G.M.); raffaele.barbagallo@unict.it (R.B.); giuseppe.bua@phd.unict.it (G.B.); 2 Department of Electric, Electronic, and Computer Engineering, University of Catania, Via Santa Sofia 64, 95125 Catania, Italy

**Keywords:** delamination, power electronics packages, FEM simulation, cohesive zone model, interfacial behavior, reliability

## Abstract

Delamination is a critical failure mode in power electronics packages that can significantly impact their reliability and performance, due to the large amounts of electrical power managed by the most recent devices which induce remarkable thermomechanical loads. The finite element (FE) simulation of this phenomenon is very challenging for the identification of the appropriate modeling tools and their subsequent calibration. In this study, we present an advanced FE modeling approach for delamination, together with fundamental guidelines to calibrate it. Considering a reference power electronics package subjected to thermomechanical loads, FE simulations with a global–local approach are proposed, also including the implementation of a bi-linear cohesive zone model (CZM) to simulate the complex interfacial behavior between the different layers of the package. A parametric study and sensitivity analysis is presented, exploring the effects of individual CZM variables on the delamination behavior, identifying the most crucial ones and accurately describing their underlying functioning. Then, this work gives valuable insights and guidelines related to advanced and aware FE simulations of delamination in power electronics packages, useful for the design and optimization of these devices to mitigate their vulnerability to thermomechanical loads.

## 1. Introduction

Power electronics packages play a crucial role in a wide range of applications, including electric vehicles, renewable energy systems, and consumer electronics.

These devices are designed to handle high power densities and operate under harsh environmental conditions, and they typically consist of multiple layers composed of metallic and ceramic materials [1]. The main components included in these systems are semiconductor dies. These are based, typically, on silicon (Si), silicon carbide (SiC), or gallium nitride (GaN). By means of solder alloys and aluminum-based wires, the dies are attached and interconnected to the underlying copper–ceramic–copper substrate, which forms an electrical circuitry on the top layer and provides a good thermal exchange on the bottom layer. In order to insulate all the electronic devices and protect the layers subjected to oxidative and corrosive phenomena, the entire system is usually encapsulated within a silica-filled epoxy resin [2].

During their operational life, power electronic packages must be able to work in different thermomechanical conditions and they are susceptible to various failure modes. Indeed, the materials used for these applications have very different thermal expansion coefficients. Moreover, very often, asymmetric geometries are adopted. Then, upon experiencing stresses due to temperature variations induced by operative working conditions, these components suffer from warpage issues [3,4]. One of the most critical failure mechanisms is delamination [5]. Delamination refers to the separation or detachment of layers at their interfaces, leading to performance degradation, compromised reliability, operational failure of electrical functions, and even catastrophic failure as the whole package breaks. Delamination can result from various factors, including mechanical stresses induced by temperature cycling, thermal expansion mismatch, and mechanical loading during operation. Understanding and predicting the behavior of delamination in power electronics packages is essential for ensuring their long-term reliability and optimal performance. Traditional experimental methods for studying delamination are time-consuming and costly and are limited by the ability to access critical regions within the package. Therefore, numerical simulations have emerged as a valuable tool to investigate and analyze delamination behavior in a more efficient and cost-effective manner. In recent years, researchers have been utilizing FE simulations to study delamination in power electronics packages and gain insights into their initiation and propagation mechanisms [6,7], despite the latter being significantly mesh dependent. In this field, the cohesive zone models (CZMs) approach has gained popularity in recent years due to its capability of modeling the complex interfacial behavior between layers. CZMs provide a phenomenological description of the fracture process by defining a class of mathematical formulations describing the traction-separation relationships along the delamination interfaces. By incorporating CZMs into FE simulations, it becomes possible to capture the complex behavior of delamination, including crack initiation, propagation, and interaction with other structural features. In the literature, CZM has been implemented to simulate the many kinds of interfaces, including some applications related to polymers [8] and bonded joints [9,10]. Regarding power electronics applications, CZM has been used in copper-to-resin adhesion [11], solder joint interface delamination [12], the bonding/debonding behavior of bond pad structures [13], the debonding process of stiff film/compliant substrate systems [14], the failure mechanisms of stretchable electronics [15], the solder layer and semiconductor chip interface [16], and thermal fatigue [17], among others. Several approaches have been proposed in order to take into account the damage for fatigue loading, such as linking it to the maximum load the cycle [18,19] or the maximum principal strain [20] or including an unloading–reloading relation [21,22]. However, the parameters of the CZM cannot be directly calibrated and there is no unique accepted method to obtain them; in many cases, they are fitted considering the experimental behavior of the component at hand [8,23,24,25].

In this study, we focus on the finite element simulation and analysis of delamination in a reference power electronics package using a bi-linear cohesive zone model. By utilizing this advanced numerical approach, we aim to enhance the understanding of delamination in power electronics packages and to provide valuable insights for design optimization and reliability assessment. The objective of this work is to assess the role of the individual CZM parameters in the delamination behavior and to understand how each parameter affects one aspect or another of the macroscopic degradation behavior. The findings will provide guidelines and recommendations for performing finite element simulations of delamination in power electronics packages, including the conscious calibration of CZM when actual experimental results are available, specifically referring to the application of power electronics, enabling more reliable and efficient design processes.

## 2. Materials and Methods

The main objective of this work is to propose an advanced modeling approach to simulate the delamination phenomenon in power electronics packages using the FE method, with a particular emphasis on accurately describing its functioning and outcomes dependent on its constitutive parameters. Indeed, per se, the proposed tools are among the most advanced and accurate modeling solutions for these kinds of applications, and their accurate implementation description is already valuable from a technical applicative point of view. Moreover, typically the modeling tools used for delamination simulations are calibrated through reverse procedures to replicate experimental results. However, there is no universally accepted methodology for this purpose. Therefore, there is a need for valuable information to comprehensively understand the functioning of these modeling tools, enabling their conscious calibration, with regard to the specific application.

**Figure 1:**
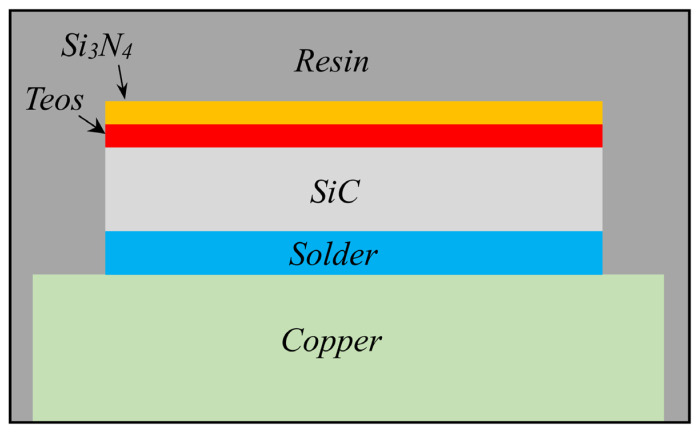
Layer sequence of the electronic package (not to scale).

**Figure 2:**
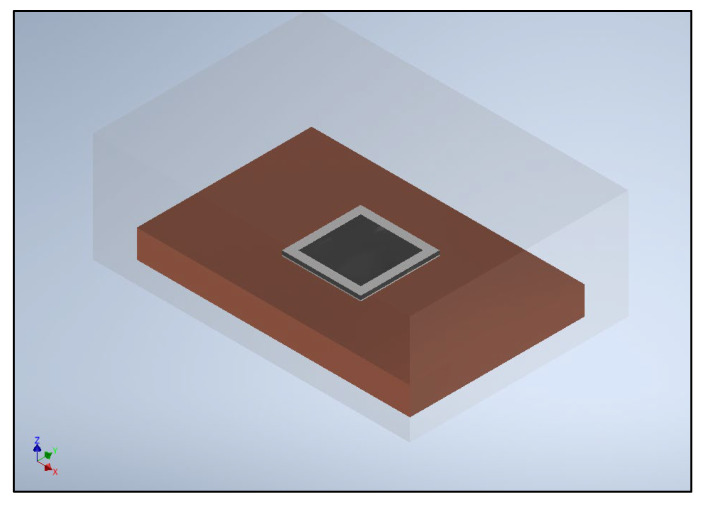
Isometric view of the power electronics package.

With this main aim, the delamination behavior of a generic power electronics package subjected to a linear law of temperature–time is herein simulated. Two main modeling choices are employed: a global–local approach and the utilization of cohesive elements to model one of the package’s critical interfaces. The representative power electronics package considered in this study adopts one of the layers layouts possible for this kind of component [26], as shown in the qualitative diagram of Figure 1. Specifically, the layers are, from bottom to top, the copper base plate; the solder layer (PbSnAg), which realizes the structural and electrical connection with the upper silicon-based layer (silicon carbide, SiC); and the following layer of TEOS (tetraethyl orthosilicate), which provides the silicon source for the successive deposed layer of silicon nitride (Si_3_N_4_). The whole multilayer package is then encapsulated in a thermosetting resin. According to the isometric view of the package in Figure 2, the resin surrounds the whole package and fills the inner central square cavity within the TEOS and SiN layers.

The material properties of each layer, as presented in Table 1, are taken from the literature [27,28,29,30,31,32]. The overall size of the package (encapsulating resin) is supposed to be 14.5 mm by 10 mm in the reference x–y plane, with a thickness along the z direction of 5 mm. The representative die has an outer square contour of 3.6 mm and the thicknesses of its layers range from the order of magnitude of 1 µm for the Si_3_N_4_ and the TEOS, to nearly 50 µm for the solder, to nearly 200 µm for the SiC.

The huge difference between the thicknesses of the different components of the package, ranging from 1 µm (Si_3_N_4_) to 5 mm (resin), clearly poses the main preliminary modeling issue.

**Figure 3:**
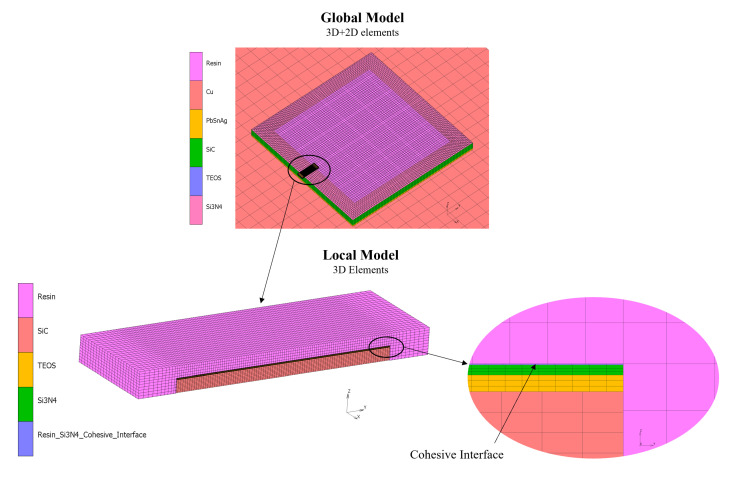
Global and local models’ relationship and details.

The package structure was meticulously modeled with the commercial software for finite elements MSC Marc 2015^®^. The study of the local delamination problem requires a strong refinement of the mesh in the areas of interest. Moreover, as mentioned above, the involved material layers exhibit very different thicknesses and plan dimensions. Therefore, given the large number of simulations to be run, a single comprehensive model with a sufficiently dense mesh would have required a great computational effort. Thus, a global–local approach was adopted. Firstly, a global analysis was performed under a linear temperature–time law on a comprehensive model with a sufficiently refined mesh to accurately describe the global deformation of the system. Then, a local sub-model encompassing a representative lamination-wise critical volume with a denser mesh was created. The nodal displacements, forces, and temperatures calculated from the global model were imposed on the nodes lying on the local domain’s frontier. The CZM was implemented in the local model to describe one of the most critical interfaces of the package, i.e., the resin/Si_3_N_4_ interface. Figure 3 shows both the global and local models with their relationship and details.

In both models, all materials were considered to be elastic, linear, and isotropic, with the characteristics shown in Table 1. The large deformations option was activated in order to take into account geometric non-linearities.

In the global model, given the micrometric thickness of the TEOS and Si_3_N_4_ layers compared to their much greater plan dimensions and to the other layers’ thicknesses, 2D thin-shell-type elements were considered for the smaller layers and 3D elements for the remaining layers. In particular, the thinnest layers of the package, together with the part of the resin which fills their internal central square cavity, were modeled with 2D Quad (4) thin-shell elements. The resin capsule was modeled with 3D Tetra (4) elements. The remaining layers were modeled with 3D Hexa (8) elements. Rigid contact was implemented at all interfaces between different materials. In particular, glued contact was implemented for the Hexa/Hexa and the Hexa/Quad interfaces, while rigid links were used to connect the shell/shell interface since 2D elements do not accept the glued contact on both of their faces. The structural constraints modeled a planar frictionless support, simulating that the package is placed over the bench of a climatic chamber and is subjected to the prescribed temperature histories, permitting the free expansion and warpage of the overall system. Specifically, one hinge and two mutually perpendicular slides on the x–y plane were imposed on three of the four lower vertex base points of the package.

In the local model, the geometry was finely discretized, paying particular attention to the interfaces between the layers. Cohesive elements were implemented to describe one of the most critical interfaces of the package in terms of delamination, i.e., the resin/Si_3_N_4_ interface. Indeed, cohesive elements were specifically designed to capture the mechanical behavior of the interfaces and provide an accurate representation of the initiation and propagation of damage. Despite being a comparative study, a mesh-dependency analysis was carried out in order to guarantee the accuracy of the CZM simulation results. For certain reference simulations, an evaluation was conducted regarding the maximum dimensions of the cohesive elements below which the numerical differences in the results compared to a very fine mesh were found to be below 1% in terms of the delaminated area. Then, this final mesh was considered for all of the remaining simulations.

According to CZM, the element reacts as a non-linear spring in which the reaction force per unit interface area, also called traction *t*, depends on the relative displacements between the upper and the lower edge of the element, also called separation *δ*. The relative displacement modulus between a single pair of integration points or nodes is obtained by the vectorial sum of one normal and two shear components. Until the separation reaches a particular value defined as the critical opening displacement (*COD*), which corresponds to a maximum stress *σ*_max_, the cohesive interface is characterized by a traction force linearly increasing with the separation. Afterwards, the damage starts to develop and causes the degradation of the stiffness *K* of the cohesive zone, in terms of an irreversible loss of stiffness (softening behavior). The element becomes fully damaged when the separation becomes equal to the maximum opening displacement (*MOD*). The cohesive element response described before, accounting for the damage initiation and progression, is expressed by the so-called traction-separation law. The integral of the traction with respect to the separation, i.e., the area beneath the traction-separation law, expresses the potential energy released per unit crack growth area in a given crack opening mode. In the fracture mechanics literature, this is also called the critical strain energy release rate or cohesive energy *Gc*.

**Figure 4:**
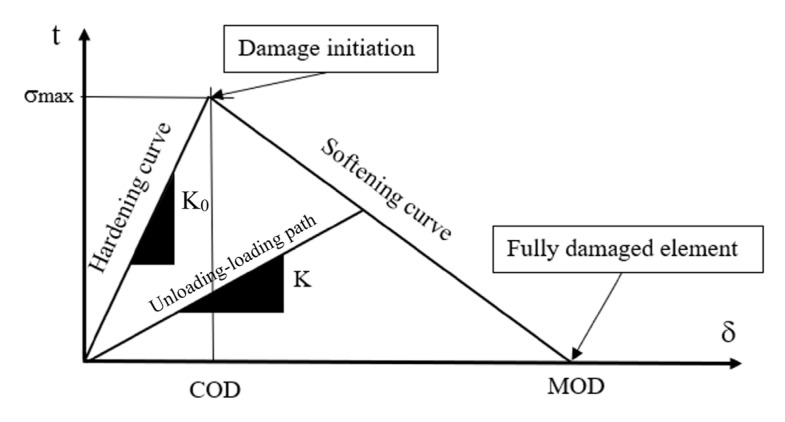
Qualitative representation of the bi-linear characteristic curve of the cohesive elements.

Thus, there are essentially three independent parameters to calibrate in CZM: *COD*, *MOD*, and *Gc*. A qualitative representation of the bi-linear triangular characteristic curve of the implemented elements is shown in Figure 4.

It is possible to describe the behavior of the cohesive zone under different load conditions such as pure traction, pure shear, and a mixed mode by using more than one traction-separation law. In this comparative analysis, the tensile and shear responses are considered to be equal to one another; thus, we used a single bi-linear traction-separation law. Equation (1) shows the mathematical formulation of the bi-linear model, where $K_{0}=\sigma_{max}/COD$ is the element’s stiffness in the elastic region, $a=\sigma_{max}/(COD-MOD),$ and $b=MOD\;\sigma_{max}/(MOD-COD)$.

(1) $$t\left(\delta \right)=\left\{\begin{array}{c} K_{0}\delta \;if\;\delta \;\leq \;COD\\ a\delta +b\;if\;\delta \;>\;COD \end{array}\right.$$

In this case, it is immediately possible to evaluate the cohesive energy *Gc* by using the formula for the triangle’s area (Equation (2)). Indeed, the triangle’s height is equal to *σ_max_* and the base is equal to the *MOD*.

(2) $$G_{c}=\frac{1}{2}\;\sigma_{max}\cdot MOD$$

The law presents an initial linear reversible section, during which the interface’s stiffness *K*_0_ remains constant, followed by a linear irreversible response during which the stiffness gradually decreases as the damage evolves according to Equation (3).

(3) $$D=1-\frac{K}{K_{0}}$$

*K* is the element’s stiffness during the unloading/re-loading transformation, which starts from a point lying in the softening curve (Figure 4). When *δ* reaches the *COD* value, the stiffness is still equal to the elastic hardening region value and the damage variable is equal to zero. When *δ* reaches the *MOD* value, the element’s stiffness degenerates to zero and the cohesive element is fully damaged (*D* = 1).

## 3. Results and Discussion

The global model of the package is subjected to a linear time-variable temperature law, uniform on all points, between −65 and 150 °C which represent realistic temperatures for the passive testing of this kind of application. The differences between the materials’ thermal characteristics induce corresponding different deformations in the layers and, in turn, out-of-plane displacements with an overall warpage of the package. Thus, the nodal displacements and temperatures calculated from the global model are imposed on the nodes lying on the external interface of the local model. Several simulations of the local model were carried out, changing the characteristics of the cohesive elements representing the resin/Si_3_N_4_ interface in order to evaluate the influence of the single cohesive variables on the delamination phenomenon.

It is important to remark that the proposed study has only a comparative purpose and not an absolute value, due to the highly mesh-dependent phenomenon under analysis. Thus, the aim is to compare the behavior of different cohesive elements, depending on their characteristic parameters, under realistic thermomechanical conditions for power electronics applications.

**Figure 5:**
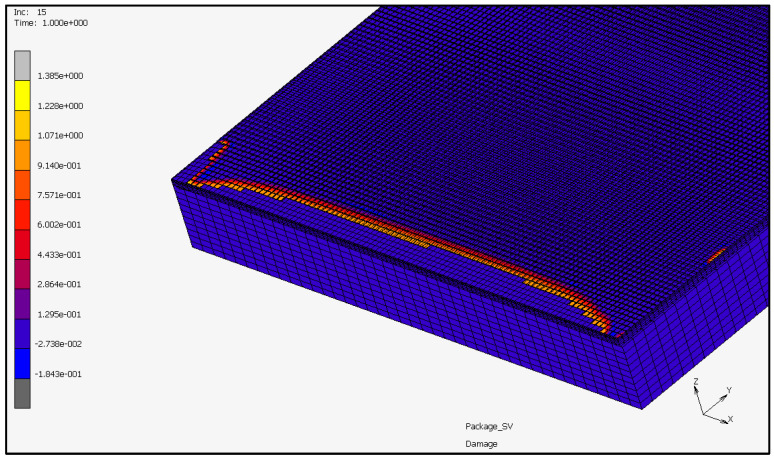
Example of simulation output with delaminated elements and damage plot.

During the local simulations, at every step and for every cohesive element, the damage variable is calculated depending on the displacement of the corresponding nodes. Once the damage variable reaches the unit value, the element is deactivated. The effect of the cohesive elements’ characteristics on the reliability of the package is measured in terms of the percentage of cohesive elements delaminated at the end of the simulation. An example of the output from the local model is shown in Figure 5, in which the damage variable is presented as a model plot. The fully damaged cohesive elements have been automatically removed and the current crack front at the given analysis increment is identified by the cohesive elements not yet removed from the model exhibiting a damage value close to one (yellow band).

The three characterizing parameters of the cohesive elements which have been varied between the different local simulations are the *MOD*, the *COD*, and the *Gc*. The value sets of these parameters used in this comparative sensitivity analysis are reported in Table 2, together with the consequent maximum traction *σ*_max_.

**Figure 6:**
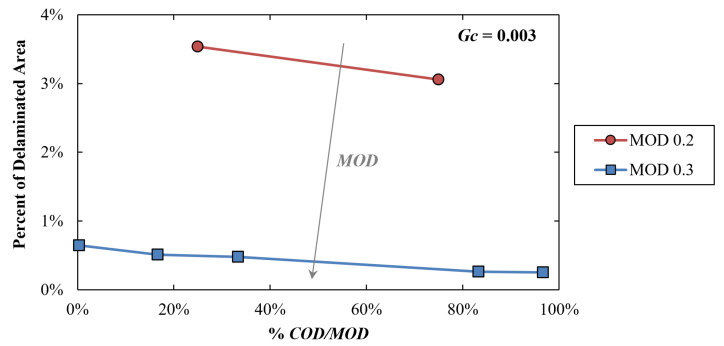
Local simulations results: percentage of delaminated area versus *%COD/MOD* with the curves parametrized with the *MOD*. *Gc* is equal to 0.003 in all simulations.

Looking at the simulations results, Figure 6 shows the influence of the ratio of *COD/MOD* on the delaminated area (percent over the total contact area) at the end of the prescribed load case (accomplished temperature variation from −65 to 150 °C). In all of these simulations, the cohesive elements are characterized by the same *Gc* value of 0.003. The curves are parametrized with the *MOD.* Thus, a single curve of the graph refers to cohesive traction-separation triangles with the same area (energy *Gc*) and height (maximum traction *σ*_max_), but different positions of the upper vertex along to the base of the triangle. It is possible to see that the %*COD/MOD* has an almost negligible effect on the delamination. Indeed, the percentage of delaminated elements only slightly decreases with the *COD* converging to the *MOD*, i.e., with the hardening recoverable part of the cohesive law growing with respect to the softening irreversible part. As expected, curves characterized by higher values of *MOD* induced a lower delamination because a greater relative displacement was needed to obtain the unit damage value of the cohesive elements.

**Figure 7:**
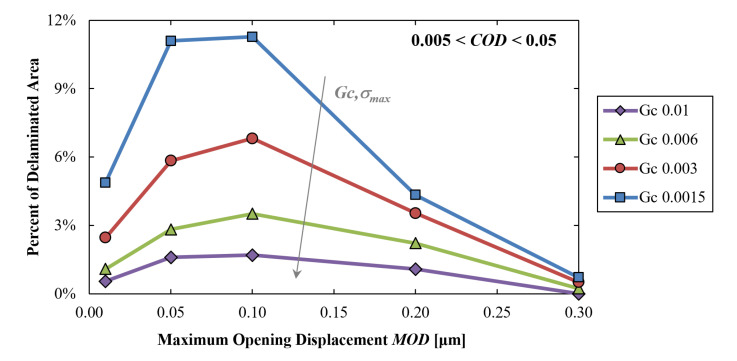
Local simulations results: percentage of delaminated area versus *MOD* with the curves parametrized with the *Gc*. *COD* is between 0.005 and 0.05 in all simulations.

Figure 7 shows the influence of the *MOD* on the delaminated area. Given its negligible influence, *COD* is set between 0.005 µm and 0.05 µm in all simulations. The curves, parametrized with *Gc*, are characterized by a somehow common trend with a peak of delamination for *MOD* around 0.1 µm and a progressively extinguishing delamination as the *MOD* approaches 0.3 µm. Instead, the delaminated area at very low values of *MOD* exhibits certain differences depending on the specific energy *Gc*. Moreover, for a given value of *MOD*, the delaminated percent area increases with decreasing *Gc.*

Going from the highest to lowest values of *MOD*, it is clear that the percentage of the delaminated area increases, since a lower relative displacement between the nodes is necessary to reach complete damage of the element. On the other hand, approaching values of *MOD* closer to zero, the counter-intuitive trend inversion which occurs for a value of approximately 0.01 µm is explained by the fact that, having fixed the value of the other variables, the lower the *MOD* value, the higher the cohesive triangle will be (greater maximum traction *σ*_max_). Thus, below a certain value of *MOD*, the traction produced by the cohesive elements becomes relevant in limiting the deformation of the elements themselves and, in turn, reducing the percentage of delamination. A hypothetical *MOD* equal to zero would induce infinite traction, i.e., no delamination.

In other words, at *MOD* values below 0.1 µm, the delamination is mostly force-driven, while at higher *MOD* values, it progressively becomes mostly displacement-driven.

The fracture of fragile interfaces (failing at smaller *MOD* values) is also driven by local forces and thus by the cohesive energy (fracture directly affected by the whole set of cohesive parameters and curves quite different from each other at *MOD* values below 0.1 µm); in contrast, the fracture of compliant interfaces (failing at large *MOD* values) is mainly displacement-driven as it only depends on the local opening value and is mostly unaffected by the other cohesive parameters (curves progressively overlapping to each other as *MOD* values tend to 0.3 µm).

Due to the thermomechanical deformation mode of interest (warpage due to temperature variation), the overall structural stiffness of the package may also affect the failure for a given set of cohesive parameters. In fact, for very stiff structures (low local displacements with forces at the interface limited by the cohesive layer), the local displacements hardly reach the *MOD* value necessary for the delamination (right end of the diagram in Figure 7, poor or not delamination at all, independent of the other cohesive parameters $\sigma_{max}$ and *Gc*).

Instead, intermediate-stiffness structures (lower forces and larger local displacements at the interfaces) can easily induce local displacements comparable to the *MOD* value with forces comparable to $\sigma_{max}$: in this case, the delaminated area increases (peak zones in Figure 7) and greatly depends on the other cohesive parameters $\sigma_{max}$ and *Gc* as well.

If the structure becomes too compliant (too low forces for inducing the displacements comparable to the *MOD*), then the amount of delamination decreases again because the local forces are lower than the cohesive traction $\sigma_{max}$, and thus the damage cannot even initiate (left side of Figure 7). In addition, in this case, the amount of delaminated area depends on both *MOD* and $\sigma_{max}$.

**Figure 8:**
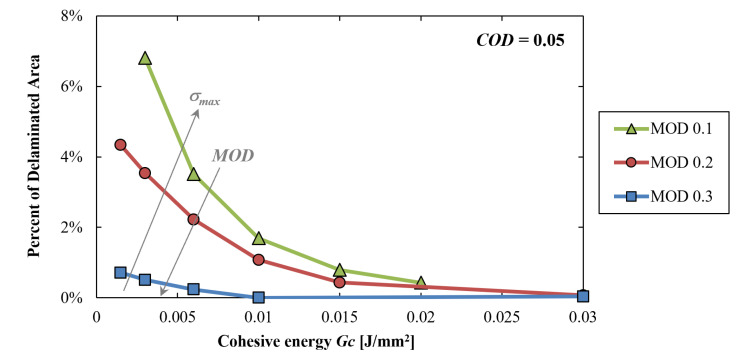
Local simulations results: percentage of delaminated area versus *Gc* with the curves parametrized with the *MOD*. *COD* is equal to 0.05 in all simulations.

Finally, Figure 8 shows the influence of *Gc* on the delamination. *COD* is set to 0.05 µm in all simulations. The curves parametrized by the *MOD* exhibit a common monotonic trend. Indeed, the greater the *Gc*, the lower the delamination. Moreover, curves with a lower *MOD* induce a greater delaminated area.

The general descending trend of the curves with the *Gc* is easily explained by the fact that, as already seen before, with the other variables fixed, the greater the *Gc*, the higher the cohesive triangle will be and, in turn, the greater the produced limiting delamination traction will be. However, in this case, it is possible to see that when approaching very low values of *Gc*, the parametrized curves do not tend to the same value. Indeed, even if the *Gc* becomes very low, in order to induce delamination in an element, it is still necessary that the local displacements reach the value of the *MOD*.

Summarizing the obtained results, for relatively compliant interfaces, the maximum opening displacement *(MOD)* is definitely the main parameter directly influencing the separation-driven delamination, while for stiffer and more fragile interfaces, the fracture-promoting energy *Gc* largely controls the traction-driven delamination process. The transition parameter between reversible and irreversible cohesive responses, triggered by the *COD* parameter, has modest effects on the delamination which only slightly decreases as the above transition is delayed (increasing *COD/MOD* values).

The given comprehensive description of this advanced modeling approach, including a global–local approach and the implementation of CZM, provides a complete and accurate solution for the FE simulation of delamination in power electronics and similar applications. Moreover, the modeling tools used for delamination simulations are typically calibrated through reverse procedures to replicate experimental results and there is no unique accepted methodology for this purpose; thus, an accurate explanation of the model’s functioning and an analysis of the conducted parametric study and sensitivity analysis provides, given a target experimental result, fundamental reference points and guidelines for a conscious calibration of the constitutive parameters of the model, with regard to the specific application.

Future developments of this work will implement more advanced modeling of the materials of the component’s layers, with the complete description of their elasto-plastic nonlinear behavior. Moreover, instead of considering a single monotonic thermomechanical load, cyclic loading encompassing thermal fatigue could be studied with the corresponding calibration of the cohesive interface.

## 4. Conclusions

Delamination is one of the most common modes of structural failure in power electronics packages, essential for many modern high-performance real-world applications, directly compromising their electrical operability at the same time. This work aimed to propose an advanced CZM modeling approach to simulate the delamination phenomenon in these kinds of components using the finite element method, with a particular emphasis on accurately describing the implemented modeling tools functioning and outcomes dependent on their constitutive parameters. In particular, this work presents a comparative finite element study of the delamination phenomenon, referring to a representative electronics package. A global–local approach is employed, incorporating a bi-linear CZM as the main modeling tool to describe one of the most critical interfaces, i.e., the resin/Si_3_N_4_ interface. The adopted procedure allowed us to describe the complex interfacial behavior between the critical layers of the package with three parameters which can be calibrated for the specific application. On the other hand, the global–local approach allowed us to obtain greatly detailed local mechanical responses without overloading the computational effort. Indeed, several simulations were carried out to conduct a parametric study and sensitivity analysis to evaluate in detail the effects of every cohesive parameter and of their combination on the delamination of a representative power electronic package subjected to realistic thermomechanical stresses. In particular, it was highlighted that the maximum opening displacement *(MOD)* is the parameter that directly influences the separation-driven delamination of compliant, ductile interfaces, whereas either the specific energy triggering fracture *Gc* or the maximum stress $\sigma_{max}$, control the traction-promoted delamination of stiffer, more fragile interfaces. On the other hand, it was shown that the critical opening displacement *(COD)* has no relevant effects, as the delamination only slightly decreases as the damage initiation is delayed with greater *COD/MOD* values.

The presented comprehensive description of this advanced modeling approach provides valuable insights for performing FE simulations of delamination in power electronics packages using CZM. Moreover, given that the modeling tools used for delamination simulations are typically calibrated through reverse procedures to replicate experimental results and that there is no unique accepted methodology for this purpose, accurate explanations of model’s functioning and the analysis of the parametric study and sensitivity analysis conducted here provide useful guidelines for the understanding and conscious calibration of the model. Overall, the provided information can be of help in the design and optimization of power electronics packages aimed at mitigating this structural failure mode.

## Figures and Tables

**Table 1:** Material properties of the layers.

Layer	*E* [GPa]	*ν* [ - ]	*α* [K^−1^]
Copper (Cu)	125	0.30	1.8 × 10^−5^
Solder (PbSnAg)	6	0.40	2.9 × 10^−5^
Silicon carbide (SiC)	410	0.28	4.0 × 10^−6^
Tetraethyl orthosilicate (TEOS)	59	0.25	1.0 × 10^−6^
Silicon nitride (Si_3_N_4_)	300	0.29	3.4 × 10^−6^
Resin	24	0.34	1.2 × 10^−5^

**Table 2:** Analyzed parameter sets of *MOD*, *COD*, *Gc*, and *σ*_max_.

		***Gc* [J/mm^2^]**									
		0.0015	0.003	0.006	0.01	0.015	0.02	0.03	0.06	0.09	
*MOD* [µm]	0.01	0.005	0.005	0.005	0.005						*COD* [µm] *σ*_max_ [N/mm^2^]
		300	600	1200	2000						
	0.05	0.025	0.025	0.025	0.025						
		60	120	240	400						
	0.1	0.05	0.05	0.05	0.05	0.05	0.05				
		30	60	120	200	300	400				
	0.2	0.05	0.05/0.15	0.05	0.05	0.05		0.05	0.05		
		15	30/30	60	100	150		300	600		
	0.3	0.05	0.001/0.05/0.1/0.25/0.29	0.05	0.05			0.05	0.05	0.05	
		10	20/20/20/20/20	40	66.67			200	400	600	
	0.4		0.05								
			15								
	0.5		0.05								
			12								

## Data Availability

The data presented in this study are available on request from the corresponding author.
